# Emergent stenting after intravenous thrombolysis for isolated basilar artery dissection in a patient with acute ischemic stroke: a case report

**DOI:** 10.1186/s13256-021-02675-y

**Published:** 2021-03-09

**Authors:** Toshiaki Goda, Naoki Oyama, Takanori Iwamoto, Hiroki Takai, Shunji Matsubara, Masaaki Uno, Yoshiki Yagita

**Affiliations:** 1 Department of Stroke Medicine, Kawasaki Medical School, Matsushima, Kurashiki, Okayama 577701−0192 Japan; 2 Department of Neurosurgery, Kawasaki Medical School, Okayama, Japan

**Keywords:** Basilar artery dissection, Ischemic stroke, Intravenous thrombolysis, Emergent stenting

## Abstract

**Background:**

Isolated basilar artery dissection (IBAD) is a rare but important cause of ischemic stroke. Anti-thrombotic therapy is often used to treat IBAD-related ischemic stroke, but selected cases might need more aggressive treatment. There is no previous report of emergent stenting for IBAD-related ischemic stroke after intravenous thrombolysis.

**Case presentation:**

A 53-year-old Japanese woman was admitted to our hospital with disturbance of consciousness, right hemiplegia, severe dysarthria, and total gaze paralysis. Brain magnetic resonance imaging revealed no ischemic lesion, but magnetic resonance angiography showed stenosis in the basilar artery. After initiation of intravenous thrombolysis, her neurological symptoms dramatically improved. Five hours later, however, her symptoms deteriorated again. Cerebral angiography showed IBAD. Emergent stenting was successfully performed. At 90 days after stroke onset, she had no significant disability, with a modified Rankin scale score of 1.

**Conclusions:**

Emergent stenting can be an effective treatment for patients with IBAD-related ischemic stroke who are resistant to IV-rtPA.

## Background

Isolated basilar artery (BA) dissection (IBAD) is a rare but important cause of ischemic stroke (incidence, 1/400,000/year) [1]. It is known to have a poor prognosis, with a mortality rate ranging from 10 to 78.9% [2]. Anti-thrombotic therapy is often used to treat IBAD-related ischemic stroke, but selected cases might need more aggressive treatment [3]. Although some cases of endovascular stenting for IBAD have been reported, the procedures were performed electively for progressive ischemic symptoms despite adequate anti-thrombotic therapy [2–5]. To the best of our knowledge, there is no previous report of emergent stenting for IBAD-related ischemic stroke after intravenous thrombolysis. We report a case of IBAD in a patient with acute ischemic stroke who underwent emergent stenting for neurological deterioration after intravenous thrombolysis.

## Case presentation

**Fig. 1:**
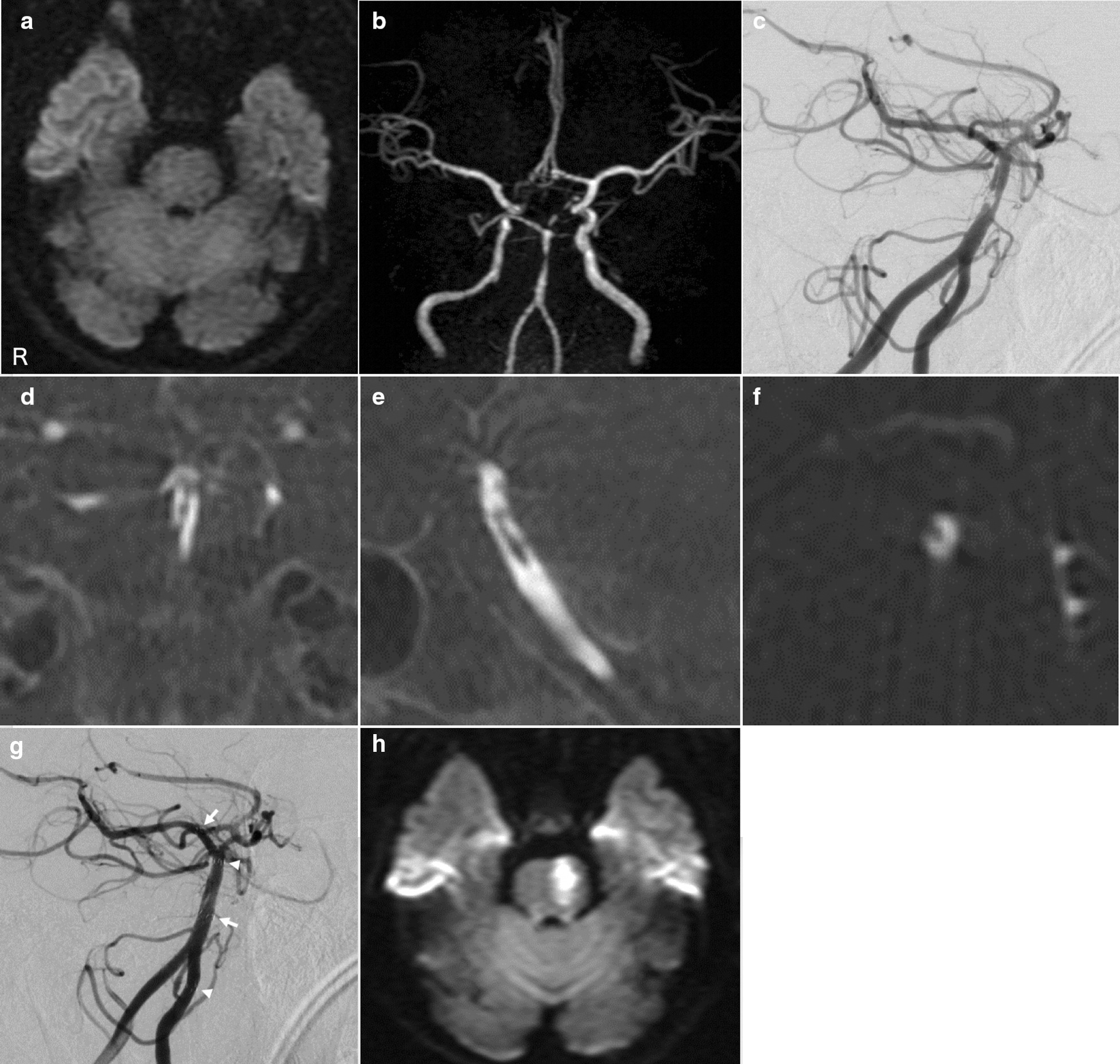
**a** Brain magnetic resonance imaging (MRI) at 90 minutes from symptom onset showing no high-intensity area on diffusion-weighted imaging (DWI). **b** Brain magnetic resonance angiography showing stenosis in the middle portion of the basilar artery (BA). **c** Cerebral angiography (45° right-anterior oblique view) showing intimal flap and double lumen in the middle and distal portions of the BA. Maximum intensity projection images of 3D rotational angiography showing double lumen of the BA in the coronal view (**d**), sagittal view (**e**), and axial view (**f**). **g** Cerebral angiography (45° right-anterior oblique view) after stenting. Enterprise VRD 4.5 × 22 mm^2^ (arrow: distal and proximal marker) and 4.5 × 28 mm^2^ (arrowhead: distal and proximal marker) was deployed from the P1 portion of the right posterior cerebral artery to the V4 portion of the left vertebral artery. **h** Brain MRI on day 6 showing left-sided pontine infarction on DWI

A 53-year-old Japanese woman with a medical history of diabetes mellitus and no other risk factors for arteriosclerosis was admitted to our hospital. Physical examination showed a Glasgow Coma Scale score of E3V3M4, right hemiplegia, severe dysarthria, and total gaze paralysis. The National Institute of Health Stroke Scale (NIHSS) score was 22. Brain magnetic resonance imaging (MRI) at 90 minutes from the onset of symptoms revealed no high-intensity area on diffusion-weighted imaging (Fig. 1a). Brain magnetic resonance angiography showed stenosis in the BA (Fig. 1b). After initiation of intravenous administration of recombinant tissue plasminogen activator (IV-rtPA), neurological symptoms improved with an NIHSS of 4; however, 5 hours after IV-rtPA, the symptoms deteriorated again with an NIHSS of 22. Cerebral angiography showed severe stenosis and double lumen in the BA (Fig. 1c–f). We deployed Enterprise Vascular Reconstruction Device (VRD) 4.5 × 22 mm^2^ and 4.5 × 28 mm^2^ (Johnson & Johnson Codman, Miami, FL, USA) from the right posterior cerebral artery to the left vertebral artery (Fig. 1g) after administration of 200 mg of aspirin and 300 mg of clopidogrel. On day 2, 100 mg/day aspirin and 75 mg/day clopidogrel were initiated. Although MRI revealed small pontine infarction (Fig. 1h), the patient’s neurological deficit gradually improved. She was transferred to the rehabilitation center on day 23 with an NIHSS of 3. At 90 days from stroke onset, she had no significant disability with an NIHSS of 0 and a modified Rankin scale score of 1.

## Discussion and conclusions

This is the first case report of a patient with acute ischemic stroke due to IBAD who underwent emergent stenting for neurological deterioration after IV-rtPA. Although some cases of stenting for IBAD have been reported, most of the procedures in these cases were performed after at least 3 days of dual antiplatelet therapy [2–5], and there is only one report of stenting in the hyper-acute phase of ischemic stroke [3]. There is no previous report of emergent stenting after IV-rtPA for IBAD-related ischemic stroke. Our case suggests that even for patients with IBAD-related ischemic stroke who are resistant to IV-rtPA, stenting can be a safe and effective treatment option.

Optimal treatment for ischemic stroke with IBAD has not been established. In clinical practice, anticoagulant or antiplatelet therapies are usually used. However, conservative management occasionally results in a poor prognosis [3, 6]. Efficacy and safety of IV-rtPA for ischemic stroke due to intracranial artery dissection have not been established, and in some cases, neurological deterioration after IV-rtPA is noted [7]. For patients presenting with progressive ischemia despite adequate medical treatments including IV-rtPA, stenting can be an alternative treatment option with a relatively good prognosis.

The benefits of stenting for IBAD are not completely understood. Occlusion of perforating branches of the BA is reported to be the main mechanism underlying IBAD-related ischemic stroke [8]. We speculate that thrombus formation in the false lumen might obstruct the blood flow in perforating branches by compressing the origin of these branches, resulting in brainstem infarction. IV-rtPA can prevent thrombus formation, but its efficacy is transient. In contrast, stenting can repair the intimal flap, which is the inflow route of the false lumen. Reduced blood flow into the false lumen will lead to less thrombus formation in this structure.

In conclusion, emergent stenting can be an effective treatment for patients with IBAD-related ischemic stroke who are resistant to IV-rtPA.

## Data Availability

Not applicable.

## Abbreviations

BA: Basilar artery
IBAD: Isolated basilar artery dissection
NIHSS: National Institute of Health Stroke Scale
MRI: Magnetic resonance imaging
IV-rtPA: Intravenous recombinant tissue plasminogen activator
DWI: Diffusion-weighted imaging
